# Why historical data matter for understanding the long-term effects of measles

**DOI:** 10.3389/ijph.2026.1610290

**Published:** 2026-09-28

**Authors:** Harris Bendel, Katarina L. Matthes

**Affiliations:** 1 Section of Health and Society, Department of People and Technology, Roskilde University, Roskilde, Denmark; 2 PandemiX – Center for Interdisciplinary Study of Pandemic Signatures, Roskilde University, Roskilde, Denmark; 3 Anthropometrics & Historical Epidemiology Group, Institute of Evolutionary Medicine, University of Zurich, Zurich, Switzerland

**Keywords:** child mortality, historical demography, historical epidemiology, immune amnesia, measles

Although measles prevention is well understood, there remains a contested area of study pertaining to how the measles virus affects the immune system and mortality in the months to years following infection [1]. Studies on the long-term consequences of measles remain essential to our understanding of the full impact of measles and the other infectious diseases it amplifies. This is particularly relevant in light of recent global outbreaks, which suggest that the WHO goal of measles elimination by 2030 is unlikely to be achieved [2]. Vaccine programs having been disrupted by the COVID-19 pandemics [3], and countries which previously attained elimination status are seeing measles reemerge due to vaccine hesitancy [4]. Measles therefore remains a potentially fatal infection with further-reaching implications for global health.

Measles is known to have a deleterious affect on the immune system, causing severe, potentially fatal acute secondary infections. However, studies on the long term affects, often referred to as “immune amnesia”, have produced inconsistent results when describing the length of immune suppression as well as its overall impact on the infectious disease mortality burden, with some suggesting that up to half of all childhood infectious disease mortality in the pre-vaccine era is related to measles immune effects [5–9]. Much of the difficulty of studying the impact of measles-induced immune amnesia stems from the inherent unequal distribution of measles infections. In an observational setting, natural measles cases in high income countries are often strongly confounded by variables such as pre-existing comorbidities, and healthcare-seeking behaviors. In the post-vaccination era, the extreme rarity of measles cases ensures that the few individuals who do contract the virus are inherently unrepresentative of the broader population, introducing significant selection bias into observational studies. Alternatively, in high burden populations where measles is more common, there persists inequalities in healthcare access which complicates the establishment of reliable cohorts. Working within these restraints, studies have shown a multi-year, increased risk in non-measles infections post-measles infection, when compared to individuals who were not sick with measles [8, 9]. However, these studies using contemporary individual data cannot link immune amnesia to mortality due to limited cohort sizes and low mortality rates. Conversely, studies focusing on mortality, that have been used to infer the overall mortality burden of measles immune amnesia, do so by comparing survival of individuals vaccinated and unvaccinated for measles. They do not track measles infections, or use cause specific mortality, and similarly are heavily affected by confounding variables [10, 11].

The claim that measles immune amnesia may have contributed to up to half of all childhood deaths from infectious diseases in the pre-vaccine era, and that following the introduction of vaccination, the subsequent decline in measles infections was the primary driver of reduced childhood infectious disease mortality comes from a paper by Mina et al. [7]. These authors measured measles immune amnesia by quantifying the drop in non-measles infectious disease mortality in three countries before and after the introduction of the measles vaccines. This statement does not address mortality fluctuations across history and regions, but in the case of Denmark, the data used to make this claim, comes from when infectious disease mortality was already nearing historic lows. In 1986, the year before the measles vaccine was introduced, there were a total of 19 deaths from non-measles childhood infections, 5 years later, in 1991, there was a total of 16 deaths. This makes the results from Mina et al. [7] unsuitable for speculating on the contribution of measles immune amnesia to childhood infectious disease death in the pre-vaccine era, because by the 1980s, many of the most significant contributors to infectious disease mortality had been largely eradicated.

This claim is also inconsistent with broad trends in historic measles infections and mortality rates. For example, in the early 20th century in Copenhagen, census data shows childhood mortality is highly concentrated towards the first year of life [12], well before the average age of measles infection which was estimated to be 5.65 years [13] using weekly measles infection numbers taken from 1907 to 1930. To visualize this, Figure 1 shows the age distribution of measles infections and the non-measles deaths per 10,000 by age. The distribution of deaths from measles and other infectious diseases comes from an annual report on epidemic diseases in Copenhagen from 1911 shown in Table 1.

**FIGURE 1:**
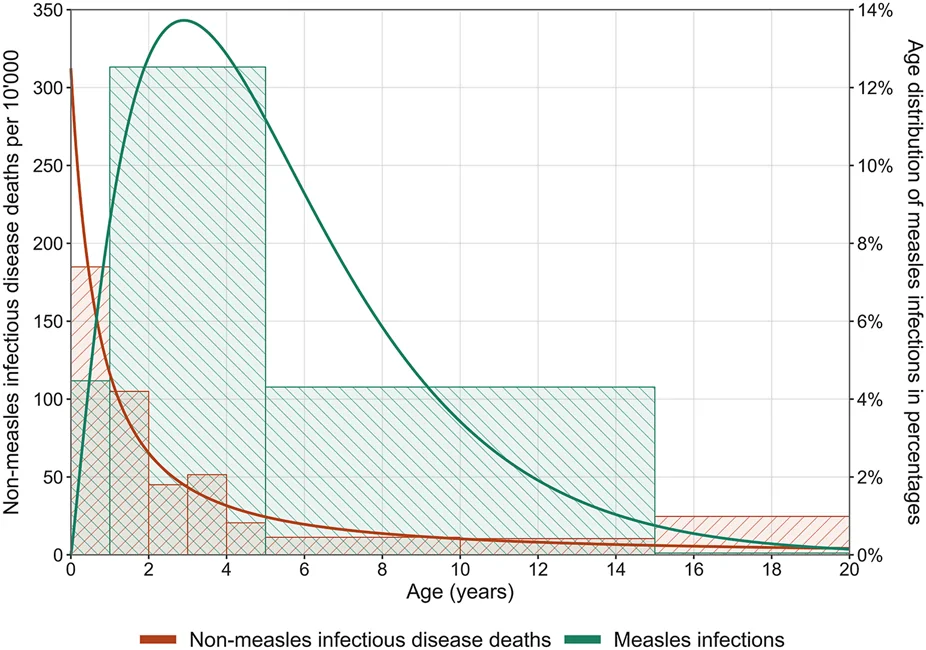
Age distribution of measles infections and non-measles infectious disease deaths (Copenhagen/ 1911). Annual counts of measles infections were reported in four age brackets; under 1, 1-5, 5-15, and 15-65. These totals were fitted to a gamma distribution to show the overall trend. Mortality data was in higher granularity with yearly frequency up until age 5, and then 5-10, 10-15, and 10-20. These totals were fitted with a power law function to show the overall trend. Notably, “infant diarrhea”, the most significant source of mortality among under 1s at 286.6 per 10,000 was not included in the death total because it is considered food and water borne.

**TABLE 1:** Deaths by cause and age groups (Copenhagen/ 1911), Source: Stadslaegens Aarsberetning.

Cause	< 1	1	2	3	4	5–9	10–14	15–19	Total
Infant diarrhea	279	21	4	1	—	2	1	1	309
Whooping cough	95	62	10	7	1	1	—	—	176
Pulmonary phthisis	5	—	1	3	2	1	5	53	70
Scarlet fever	4	9	7	8	5	21	5	2	61
Tuberculous meningitis	8	6	7	8	2	6	6	—	43
Measles	14	17	3	2	2	2	—	—	40
Tuberculosis, other sites	6	2	3	1	1	3	5	16	37
Tuberculosis	6	7	2	8	2	1	4	6	36
Diphtheria	4	3	5	5	5	7	4	—	33
Congenital syphilis	29	—	—	—	—	—	—	—	29
Other epidemic diseases	5	2	2	3	—	3	3	1	19
Croup	2	5	2	3	—	1	1	—	14
Pyaemia & septicaemia	7	1	—	—	—	—	2	3	13
Scrofula	—	1	1	—	—	2	1	5	10
Acquired syphilis	—	—	1	—	—	1	3	3	8
Erysipelas	5	1	—	—	—	1	—	—	7
Influenza	4	1	1	1	—	—	—	—	7
Rheumatic fever	—	—	—	—	—	—	2	1	3
Puerperal fever	—	—	—	—	—	—	—	1	1
Typhoid fever	—	—	—	—	—	—	—	1	1
All causes	473	138	49	50	20	52	42	93	917
All causes except infant diarrhea and measles	180	100	42	47	18	48	41	92	568

A fitted gamma and power-law function were used to visualize the overall trend. The trend produced by measles infection counts were similar to those produced by simulations from Metcalf et al. [13]. The lack of overlap between the distributions makes it unlikely that measles immune amnesia was a significant contributor to infectious disease mortality in 20th historic Copenhagen, but more research needs to be done on studying infectious disease mortality at the individual level to better understand its impact on the pre-vaccine infectious disease landscape.

We propose that historic data offers a unique set of advantages for studying how measles infections affect the immune system and when estimating the overall impact of measles immune amnesia on historic and contemporary mortality. Data from countries like Denmark and Switzerland have well-preserved, individual-level historic mortality data and morbidity data from a pre-vaccination period long before the first vaccine was introduced. Data from an era which mortality was still high offers a valuable opportunity to investigate how measles infections increase susceptibility to other infectious diseases and increase mortality post-infection. We are, of course, aware that analyzing measles dynamics through pre-vaccination historical data presents a distinct set of methodological challenges. Due to the pathogen’s very high transmissibility, infection was almost universal among individuals who lived long enough. Consequently, although the infected cohorts are highly representative of the general population, the ubiquity of the disease makes it impossible to establish a reliable control group of individuals who were not exposed to the virus. However, because the timing of historical measles outbreaks has been well documented through historical demographic research, study designs analogous to those conducted using contemporary data can also be reconstructed for historical populations. Moreover, detailed historical measles case and hospital records often remain available in the archives and can be linked to death certificates, enabling individual-level analyses of the long-term consequences of measles infection.

Moreover, an added benefit of studying historical data of the pre-vaccine era, is that it de-couples it from the theory commonly referred to as “non-specific vaccine effects” (NSE) which states that live attenuated vaccines provide non-specific benefits to the immune system which decrease all-cause mortality in vaccinated individuals [14]. Although the theory of NSE is not widely accepted, it exists within this discourse as an alternative explanation for the correlation between measles suppression and reduced all-cause mortality. By studying the association of measles infections and non-measles mortality prior to the availability of measles vaccines, we can more confidently attribute the effect to measles immune suppression, rather than NSE.

Taken together, these considerations illustrate the importance of historical demography and historical epidemiology and highlight the value of international networks such as the COST Action “CA22116 - The Great Leap. Multidisciplinary approaches to health inequalities, 1800–2022” [15]. The COST Action Great Leap makes individual-level cause-specific mortality data more widely available for historical populations and supports the harmonisation and comparability of historical causes of death data across countries and time periods. Such efforts are particularly important because contemporary data alone are often insufficient to understand the long-term consequences of infectious diseases such as measles and the phenomenon of immune amnesia. Even studies directly comparing vaccinated and unvaccinated children are often affected by substantial confounding, as the two groups may differ systematically with respect to socio-economic status, access to healthcare, health-seeking behavior, and parental attitudes towards healthcare and vaccination.

These limitations point to a broader research opportunity. We therefore advocate for a more systematic integration of historical demographic and historical epidemiological data into research on the long-term consequences of infectious diseases, as illustrated in this commentary by the case of measles-induced immune amnesia. Linking individual-level historical infection records with cause-specific mortality data could help overcome some of the limitations of contemporary studies and open new opportunities for investigating how infections affect subsequent health and mortality. Unlocking the potential of historical mortality and morbidity data may fundamentally expand our ability to understand how infectious diseases shape health and mortality long after the acute infection has passed.
